# Placenta increta causing hemoperitoneum in the 26th week of pregnancy: a case report

**DOI:** 10.1186/1752-1947-4-412

**Published:** 2010-12-22

**Authors:** Gentian Vyshka, Nuredin Çapari, Elmas Shaqiri

**Affiliations:** 1 Biomedical & Experimental Department, Faculty of Medicine, Rr. Dibrës 371, Tirana, Albania; 2 District Obstetrical Hospital, Saranda, Albania; 3 Department of Forensic Pathology, Institute of Legal Medicine, Rr. Dibrës 372, Tirana, Albania

## Abstract

**Introduction:**

Placenta increta is a serious complication of pregnancy. We describe a case leading to uterine rupture associated with massive intra-abdominal hemorrhage.

**Case presentation:**

A 34-year-old Caucasian Albanian woman, gravida 2, para 1, was admitted to the emergency department of our hospital for acute abdominal pain associated with profound secondary anemia. An anatomopathological diagnosis of placenta increta destruens was made. An urgent hysterectomy was performed after resuscitation procedures, applied due to the severe anemia and the abdominal drama accompanying the case. Intra-operatively, a uterus-saving procedure was found to be impossible, and hysterectomy remained the only surgical option. The uterine structures were sent for further microscopic evaluation. On histological examination, deep trophoblastic infiltration of the uterine wall was observed, justifying the surgeon's decision. Our patient received blood transfusions and antibiotics. Her sutures were removed on the eighth postoperative day and she was discharged the following day in a stable condition.

**Conclusion:**

This case, describing a patient with uterine rupture and massive hemorrhage, illustrates a serious and potentially fatal complication of placenta previa. In such cases, surgery is essential, and hysterectomy may be the only viable option.

## Introduction

Placenta increta is a serious complication of pregnancy. It is characterized by entire or partial absence of the decidua basalis, and by the incomplete development of the fibrinoid (Nitabuch's) layer. Although it is considered a rare occurrence with a prevalence of approximately 1 in 2500-7000, it is associated with high morbidity and sometimes with a lethal outcome, mainly as a result of severe bleeding, uterine rupture and infections [1].

Correlations have been suggested with placenta previa, previous uterine curettage, previous cesarean sections, multiparity (six or more pregnancies), and advanced maternal age [2]. The precise etiology of this condition remains unclear.

## Case presentation

A 34-year-old Caucasian Albanian woman was admitted during the 26th week of her second pregnancy for severe anemia and diffuse abdominal pain, and with the suspicion of uterine rupture. She had given birth 14 years previously to a healthy child by caesarean section. Her medical history included no other episodes of surgery and no internal disease. This second pregnancy was considered normal by the family obstetrician; two months before her urgent admission, our patient had undergone routine sonography, which had given normal results.

Upon admission, our patient had profound anemia with a red blood cell count of 1.71 × 10^6^/mL (normal 4.-6.2 × 10^6^/mL) and hemoglobin of 5.6 g/dL (normal 11.5-16.5 g/dL). No fetal sounds could be heard, and the overall state of our patient was deeply compromised because of the acute and painful abdominal process. Following resuscitation she was sent for emergency surgery

**Figure 1:**
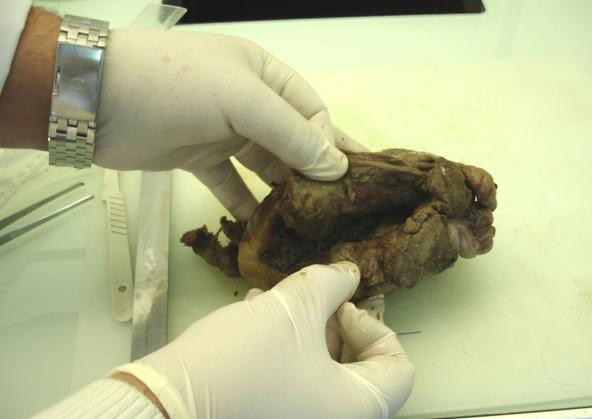
**Wide uterine rupture**.

A midline laparotomy was urgently performed, and approximately 1800 mL of intra-abdominal blood was drained. A rupture was detected at the left superior angle of the uterus; the fetus was dead, and was still implanted inside the uterine cavity. The fetus was removed through the wide rupture line (Figure 1). Intra-operatively, it was considered impossible to save the uterus, especially in terms of another possible pregnancy. Because the quantity of intra-abdominal blood removed was considerable and the uterine rupture was considered sufficiently large to prevent a uterus-saving procedure, the surgeon opted not to use an arterial ligature.

**Figure 2:**
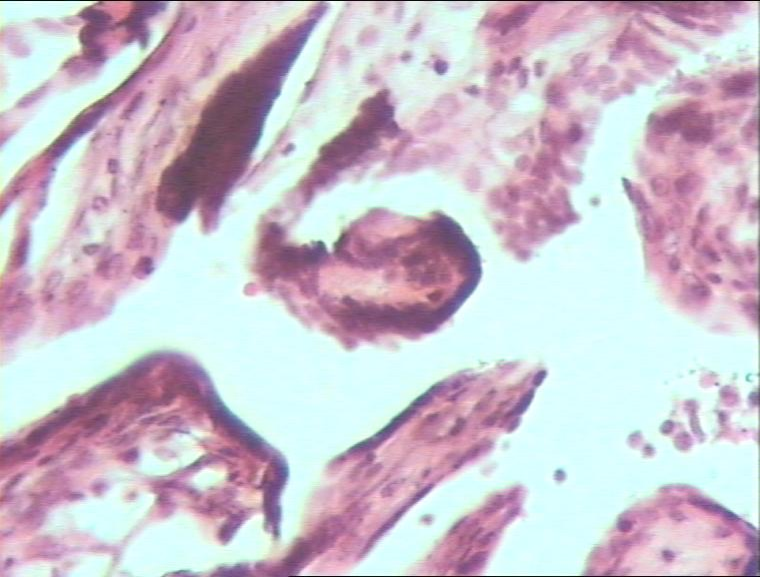
**Trophoblastic islands in the myometrium (haematoxylin and eosin, original magnification × 40)**.

**Figure 3:**
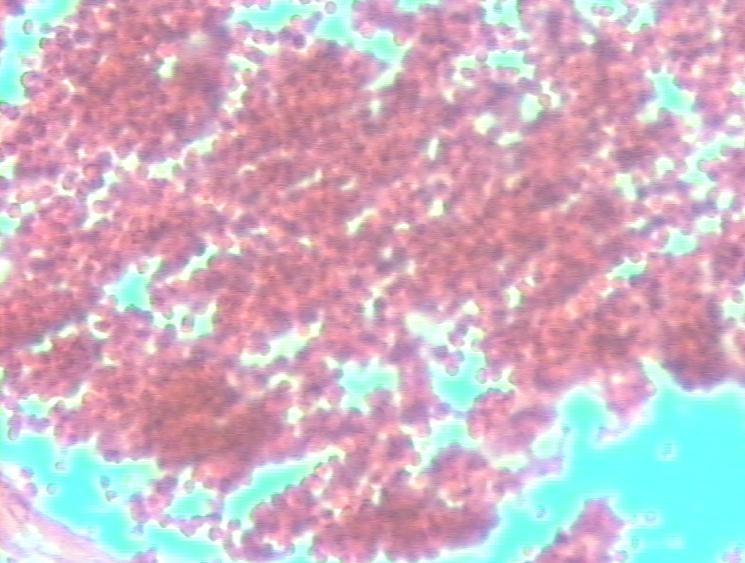
**Fresh red blood cells in the rupture line of the uterine wall (haematoxylin and eosin, original magnification × 160)**.

**Figure 4:**
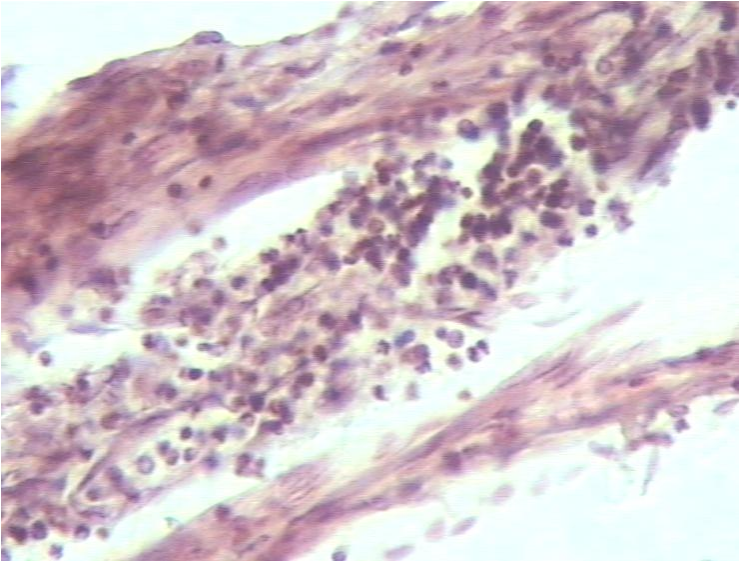
**Polymorphonuclear lymphocytes infiltrating the uterine wall (haematoxylin and eosin, original magnification × 160)**.

The uterine tissue was sent for pathologic evaluation. Trophoblastic islands were found inside the myometrium (Figure 2). Fresh red blood cells were found in the rupture line (Figure 3). Progressive and aggressive infiltrates of polymorphonuclear lymphocytes were also found inside the uterine wall (Figure 4).

Our patient received a transfusion of four units of fresh whole blood (group A, Rh positive), along with saline perfusions and antibiotics. Two weeks after the hysterectomy, her red blood cell count was 3.6 × 10^6^/ml, and the hemoglobin level 11.2 g/dl. The sutures were removed on the eighth postoperative day, and she was discharged the next day in a stable situation.

## Discussion

The clinical features of placenta increta, such as hemorrhage, uterine rupture and inversion, and invasion of the urinary bladder, are all related to the site of placental implantation, the depth of myometrial invasion, and the width of abnormally adherent placental tissue [3]. Myometrial invasion of trophoblastic islands at the site of a previous cesarean section may cause uterine rupture long before the onset of delivery, as in our patient.

In these cases, resuscitation procedures (when appropriate) and an urgent hysterectomy seem to be the treatment of choice. Previously, a more conservative treatment, aiming at uterine rescue, was followed, based upon manual removal of as much placental tissue as possible. Fox *et al*. reported that 25% of the women died during this treatment [4]. Under these circumstances, the more conservative treatment can be achieved only in cases of a partial placenta accreta/increta, when bleeding is minimal. Alternative interventions include ligature of uterine artery or internal iliac artery, or angiographic embolization [5].

There are a number of risk factors leading to hemoperitoneum during pregnancy. Previous gynecological procedures, pregnancies, infections and curettage, trophoblastic disease, and endometrial or cervical malignancies favor such an occurrence [6]. Spontaneous uterine rupture may also follow adenomyosis, instrumental termination, manipulations during labor, misoprostol-induced labor, or cocaine misuse. In some cases, no cause can be identified, and these are considered idiopathic [7-9].

Prenatal diagnosis of placenta increta can be performed using Doppler sonography and magnetic resonance imaging [10]. However, the diagnostic value of sonography in prenatal diagnosis of an asymptomatic placenta increta is uncertain. Finberg *et al*. reported a positive predictive value of 78% and a negative predictive value of 94% [11], but other authors suggested that sonography might detect only around 33% of cases of placenta accreta/increta [12].

Regarding treatment, hysterectomy is probably the best option for long-term outcome, as previously reported [13]. There have been attempts to treat placenta increta with various drugs to allow the pregnancy to continue [14]. The most widely used drug is methotrexate, although its safety and the efficacy in this setting are questionable [15,16].

## Conclusion

Our patient presented with uterine rupture in the emergency department. This case illustrates a serious and potentially fatal complication of placenta increta, due to massive hemorrhage.

## Competing interests

The authors have no competing interests hereby to declare. No funds were granted to support the present study.

## Consent

Written informed consent was obtained from our patient for publication of this case report and accompanying images. A copy of the written consent is available for review by the Editor-in-Chief of this journal.

## Authors' contributions

GV wrote the paper, checked the medical records and the literature, and revised the manuscript in accordance with the reviewers suggestions. NÇ is the surgeon who performed the operation. ESH performed the pathological sections and microscopic examinations. All authors read and approved the final manuscript.
